# Guillain-Barré Syndrome Presenting as Painful Weakness and Edema of the Legs: A Case Report

**DOI:** 10.7759/cureus.40641

**Published:** 2023-06-19

**Authors:** Amteshwar Singh, Evani Jain, Venus Sharma, Amitasha Sinha, Waseem Khaliq

**Affiliations:** 1 Medicine, Johns Hopkins University School of Medicine, Baltimore, USA; 2 Internal Medicine, Dayanand Medical College and Hospital, Ludhiana, IND; 3 Internal Medicine, Icahn School of Medicine at Mount Sinai, New York, USA; 4 Hospital Medicine, Beth Israel Deaconess Medical Center, Boston, USA

**Keywords:** inflammatory neuropathy, paralysis, plasma exchange, intravenous immunoglobulins, guillain–barré syndrome

## Abstract

Guillain-Barré syndrome (GBS) is an autoimmune inflammatory polyneuropathy, which can be challenging to diagnose due to variability in the initial presenting features. Pain, flaccid paresis, motor sensory disturbance, hyporeflexia, and autonomic dysfunction are the typical manifestations, although atypical features, such as ataxia, neck stiffness, dysphagia, ophthalmoplegia, bulbar palsy, and isolated upper limb weakness, may be seen. It may also progress to fatal respiratory depression. As such, timely diagnosis and treatment are essential. We present the case of a 41-year-old man who presented with a four-day history of acute-onset bilateral lower extremity swelling, decreased motor strength, diffuse muscle pain, hyporeflexia, and absent vibratory sensation. After admission, symptoms worsened, and the patient developed new-onset swallowing difficulty and urinary retention. Neurological examination findings of hyporeflexia and flaccid paralysis, along with normal thyroid function, and the absence of cord compression on spinal MRI pointed toward the diagnosis of GBS. Nerve conduction studies (NCS) and concentric electromyography (EMG) confirmed the diagnosis. The patient was treated with intravenous immune globulin (IVIG) and eventually discharged to a rehabilitation facility after a 12-day hospital stay. Later, the patient developed contractures and chronic pain consistent with post-GBS syndrome, for which we referred him for pain management and physical therapy. A rapidly progressive weakness with autonomic dysfunction should prompt suspicion of GBS and should be treated with intravenous immunoglobulins or plasma exchange without further delay.

## Introduction

Guillain-Barré syndrome (GBS) is an immune-mediated inflammatory peripheral neuropathy characterized by acute progressive flaccid paralysis and areflexia, which may or may not be associated with pain and autonomic dysfunction [1]. Most cases are reported to have a preceding history of infection, although not necessarily. The heterogeneous presentation of this disease with atypical as well as atypical clinical features may make the diagnosis challenging to clinch early. Once diagnosed, the autoimmune pathogenesis of GBS permits immune-directed therapies as treatment options [2]. We report a case of GBS presenting with acute generalized painful weakness and electrolyte derangements, causing a diagnostic dilemma.

## Case presentation

A 41-year-old man with a history of hypertension and chronic low back pain presented to the emergency room (ER) complaining of acute-onset, bilateral, lower extremity generalized weakness, swelling, and diffuse muscle pain. Symptoms started four days before presentation when the patient noticed weakness in his lower extremities. The next day, he also noticed generalized painful swelling in the legs, which came on abruptly, involving the thighs down to the feet. The symptoms of pain, weakness, and swelling progressed, so he could not bear weight and became chair bound. Prior to the onset of these symptoms four days ago, he was ambulating without any weakness or pain. He rated his pain as 10 out of 10. The painful lower extremity swelling was associated with a generalized cold sensation, exacerbated by minimal movements and light touch. Four days before the ER visit, his primary care physician prescribed furosemide 20 mg daily to help with leg edema without much relief. His symptoms continued to worsen with new weakness in his hands and arms with an inability to form a hand grip, causing him to lift his arms to grasp objects. A review of symptoms was unremarkable, including chest pain, palpitations, fever, chills, abdominal pain, nausea, vomiting, diarrhea, or any recent illness. Before the current presentation, the patient was taking lisinopril 10 mg daily, gabapentin 800 mg every six hours, and oxycodone 10 mg every four hours as needed for pain for the last two years. His surgical history was significant for L3-L4 microdiscectomy and L5-S1 fusion surgery more than a decade ago. His social history includes alcohol use disorder, but he was abstinent for three years.

On presentation, he was afebrile (36.9° C), hypertensive (blood pressure of 181/99 mm of Hg), tachycardic (heart rate 118 per minute), and breathing 17 per minute, maintaining 100% oxygen saturation on room air. He was quite distressed and in tears due to the pain (rated as 10 out of 10 on the visual analog scale). Physical exam demonstrated bilateral lower extremity edema extending up to the sacral region, decreased motor strength in bilateral upper extremities (4 out of 5 at shoulder and elbow level, 3 out of 5 at wrist level) and lower extremities (4 out of 5 at hip level, 3 out of 5 at knee and foot level) bilaterally, hyporeflexia (at biceps, brachioradialis, triceps, patella, with bilateral downward plantar response), an absent vibratory sensation at hallucis, finger, and ankle joints. Gait and coordination evaluation was limited due to generalized limb weakness. There were no signs of trauma, lesion, blistering, wounds, erythema, lymphangitic streaking, or lymphadenopathy on the lower limbs.

Initial laboratory testing was notable for hyponatremia (sodium 127 mmol/L; reference range 136-145) and hypokalemia (potassium 2.9 mmol/L; reference range 3.5 to 5.1). Rapid polymerase-chain-reaction testing for severe acute respiratory syndrome coronavirus 2 (SARS-CoV-2) was negative. EKG showed sinus tachycardia with normal axes, intervals, and wave morphologies; chest X-ray and computed tomography (CT) angiogram reported no acute abnormality. An abdominal ultrasound was unrevealing for ascites. Transthoracic echocardiogram showed normal left ventricular ejection fraction and no pericardial effusion. A normal thyroid stimulating hormone (TSH) level of 3.57 (ref range 0.36 to 3.74 mIU/mL) and normal free T4 level of 1.43 (ref range: 0.5 to 1.8 ng/dL).

On hospital day one, we corrected his serum sodium and potassium with normal saline infusion and oral supplementation, respectively. Electrolyte derangements were attributed to the recent initiation of furosemide, and severe hypokalemia was thought to manifest as periodic paresis and pain. Despite the correction of electrolyte derangements, his paresis and pain persisted, necessitating further workup. His D-dimer was elevated at 3.33 (reference range 0 to 0.49 mg/L) so an ultrasound of his lower extremities was done, which ruled out deep vein thrombosis. Differential diagnoses of hyporeflexia with motor weakness at this point included myopathy, myositis (due to an inflammatory disorder, toxic due to medication adverse effects or post-infectious), and acute motor-sensory polyneuropathy. Mildly elevated creatine kinase (CK) at 461 (reference range 35-232 units/liter) and aldolase 11.2 (reference range 0-8.1 units/liter) could not explain a primary myopathic process. Inflammatory markers were also mildly elevated, not suggestive of primary myositis process: C-reactive protein (CRP) was 3.36 (reference range <0.29 mg/dL), and erythrocyte sedimentation rate (ESR) was 32 (reference range 0-15 mm/hour). An autoimmune myositis process typically involves the proximal extremities and spares the distal extremities. Our patient had global proximal and distal extremity weakness. These findings, along with only mildly elevated inflammatory markers, made an autoimmune process less likely. The patient's neurologic exam did not show any findings localizing to the central nervous system or any typical upper motor neuron lesions (such as spastic paresis or hyperreflexia). So, dedicated brain imaging was not considered necessary at this stage, as a stroke or brain lesion would not explain the examination findings.

After ruling out myopathy, the working differential diagnosis for acute motor sensory axonal polyneuropathy pattern shifted to neurological causes such as Guillain-Barré syndrome (GBS) and metabolic causes (such as vitamin deficiencies). Given his prior history of an alcohol use disorder, B1 (thiamine deficiency; beri beri disease), B9 (folate), and B12 (riboflavin) deficiencies were considered. On hospital day two, we noticed that his vitamin B1 was low at 48 (78-185 nmol/liter), and folate was low at 2.4 (normal level >4.8 ng/ml). Homocysteine level was high (32.1; reference range 0-12.2 mmol/liter), and the methylmalonic acid level was normal at 187 (reference range 45-325 nmol/liter), both diagnostic of folate deficiency. Vitamin D levels were low (level 12, reference range 30-100 ng/ml), whereas vitamins B3 (niacin), B12, and E levels were adequate. We initiated the patient on thiamine, folate, and ergocalciferol supplements. Borrelia burgdorferi antibody (Lyme disease), human immunodeficiency virus (HIV), and hepatitis B and C infection screening were unremarkable. Urine porphobilinogen (for porphyria testing) and blood arsenic levels (for heavy metal poisoning) were also unrevealing.

**Figure 1:**
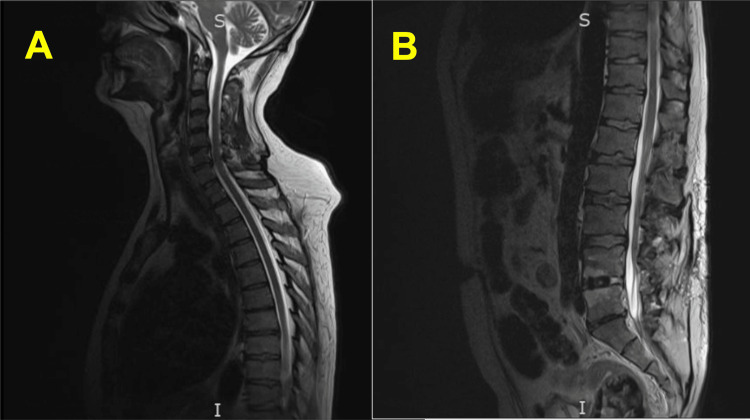
MRI (Sagittal) of the (A) Cervical and Thoracic Spine and (B) Lumbar Spine No evidence of cord compression in the spinal cord. Signal intensity in the spinal cord is normal.

On hospital day three, muscle pain worsened despite escalating doses of parenteral opioid analgesics, and the patient developed new-onset swallowing difficulty and urinary retention. His plantar reflex was absent, and his sensation to light touch was reduced globally. At this point, non-contrast magnetic resonance imaging (MRI) of the entire spine was done, which showed disc bulging at C5-6 and C6-7 with only mild central cervical canal narrowing, no significant cervical foraminal stenoses, postoperative changes with fusion at L4, L5, and S1, minimal disc bulging at L3-4 indenting the thecal sac, only mild lumbar foraminal narrowing without nerve root impingement, but no evidence of cord compression or cord signal abnormality, essentially ruling out a primary spinal cord pathology (Figure 1).

The negative inspiratory force (NIF) test was normal, suggesting no respiratory muscle involvement at that point. Due to worsening motor weakness and myopathic process being ruled out, Guillain-Barré syndrome (GBS) became the primary working diagnosis. Nerve conduction studies (NCS) revealed severe reduction in compound muscle action potential (CMAP) amplitudes except for bilateral peroneal responses, which were absent. All conduction velocities, distal latencies, and F waves latencies were normal, and all sensory responses were absent except for the right radial, which showed a reduced amplitude with preserved conduction velocity. Concentric electromyography (EMG) of select muscles showed abnormal spontaneous activity with fibrillations and positive sharp waves with absent motor unit potentials in some muscles and reduced recruitment in others. These results were diagnostic of severe sensorimotor axonal neuropathy, solidifying the diagnosis of GBS. The patient declined lumbar puncture testing, which was needed to obtain a cerebrospinal fluid (CSF) specimen and to further investigate for cytoalbuminologic dissociation. Serum testing for other immune processes was unrevealing (including negative Anti-Hu, Anti-Gm1, Anti-Gad, and anti-nuclear antibodies). Serum electrophoresis was ordered to rule out multiple myeloma as a potential differential diagnosis for spinal cord compression.

The patient was started on intravenous immune globulin (IVIG) for five days to arrest the progression of symptoms and prevent diaphragmatic paralysis. The patient was transferred to the intensive care unit (ICU) for close monitoring of neurological examination, serial NIF checks, and continuous cardiorespiratory monitoring. His motor weakness stabilized after IVIG treatment. For urinary retention, he initially needed a Foley catheter but later was able to void successfully. He was eventually discharged to a rehabilitation facility after a 12-day hospital stay. At the time of discharge, he was on acetaminophen, naproxen, oxycodone, cyclobenzaprine, nortriptyline, and gabapentin. After completing a 105-day rehabilitation, he was discharged home. He was readmitted to the hospital within three days of discharge for ongoing pain in his lower extremities, which he couldn’t manage with his analgesics. He was noted to have contractures at wrist and ankle sites, which were attributed to post-GBS syndrome. He also had recurrent urinary retention, for which a suprapubic catheter was placed. For ongoing neurologic recovery, additional physical therapy was recommended. He was also referred to pain management for post-GBS syndrome therapy.

## Discussion

GBS is an immune-mediated inflammatory peripheral neuropathy characterized by acute progressive flaccid paralysis and hyporeflexia with or without autonomic dysfunction (such as loss of bowel and bladder control and pupillary dysfunction) [3]. A global incidence of one to two per 100,000 person-years is reported [4]. A global rise in cases was noted after the epidemic due to Zika virus and SARS-CoV-2 [3,5,6]. While GBS can be seen in all age groups, the incidence seems to increase with age, affecting men more frequently than women [3]. A history of infection (respiratory or gastrointestinal) two weeks prior is often a precursor of the onset of GBS [7]. It is proposed that molecular mimicry between the peripheral nerves and microbiologic antigens is crucial to the pathogenesis of GBS. Production of autoantibodies causes the stripping of myelin from the nerves, thus producing neurological symptoms [6].

Diagnosis of GBS hinges on the patient's history, thorough neurological examination, electrophysiological studies, and cerebrospinal fluid testing [3]. Given the heterogeneity in presentation, it is necessary to rule out potential differential diagnoses and guide further diagnostic evaluation (Table 1) [3].

**Table 1:** Differential Diagnosis of Painful and Painless Paresis

A) PAINFUL PARESIS	
VASCULAR	
Abdominal Aortic Aneurysm (AAA) Rupture	It can present with loss of pulses and ischemia. Abdominal pain is typically associated with the disease. Signs of shock or sudden blood loss.
Venous Occlusion	Sudden in onset. Signs of severe ischemia such as mottling, cyanosis, or pallor. Venous thrombosis - While walking, there is leg pain and swelling; pain is relieved by extremity elevation, which distinguishes this from arterial insufficiency. May Thurner syndrome - also known as Iliac vein compression syndrome. Affects blood vessels in the legs. Can cause DVT in the left leg. The right iliac artery squeezes the left iliac vein when they cross each other in the pelvis. Therefore, the blood cannot flow freely through the left Iliac vein because of the pressure.
Arterial Dissection	History of instrumentation/high blood pressure. Sudden-onset severe pain and signs of shock.
Leriche Syndrome	Also called aortoiliac occlusive disease (AIOD). Results from atherosclerosis affecting the distal abdominal aorta, iliac arteries, and femoropopliteal vessels. Presents as a triad of lower limb claudication, erectile dysfunction, and absent femoral pulses.
External Compression/Compartment Syndrome	History of lower extremity trauma or crush injury. 5Ps – Pain, Pallor, Poikilothermia, Paresthesia, and Pulselessness.
NEUROLOGIC	
Guillain Barre Syndrome	Also known as acute inflammatory demyelinating polyneuropathy precipitated by viral infections like Cytomegalovirus, Epstein Barr virus, hepatitis A & B. Ascending bilateral leg paralysis.
Lumbar Canal Stenosis (Pseudo-Claudication)	Pain usually occurs in the morning and is unrelieved by short resting periods. Pain is relieved by leaning forward against a solid surface or by sitting.
Anterior Horn Cell	Poliomyelitis or West Nile virus infection.
Spinal Radiculopathy	Localized back pain, numbness, and tingling radiating toward the lower limbs.
Peripheral neuropathy	Commonly due to underlying diabetes mellitus. Peripheral neuropathy is difficult to differentiate from intermittent claudication. It is because intermittent claudication is accompanied by skin discoloration and diminished pulses.
NEUROMUSCULAR JUNCTION	
	Myasthenia gravis botulism, Tick paralysis.
MUSCULOSKELETAL	
Metabolic/electrolyte disturbances	Muscle weakness.
Inflammatory myositis	Muscle weakness associated with B-symptoms.
Rhabdomyolysis	Preceding trauma with hemoglobinuria and muscle weakness.
Osteoarthritis/ Connective Tissue disease	Associated with arthritic pain, aggravated by particular weather patterns or movements. Rest does not relieve pain.
Muscle strain	History of heavy work Aggravated with movements.
Ligament/ Tendon injury	Inability to walk. Popping sensation when injured.
Baker's Cyst	Posterior knee pain Not relieved by rest.
Popliteal Entrapment Syndrome	Usually observed in active young people. Various abnormal anatomic configurations of the medial gastrocnemius muscle head. When the knee is at full extension, tibial pulses may disappear.
OTHERS	
Conversion Disorder	Neurological symptoms without underlying neurologic condition.
B) PAINLESS PARESIS	
Transient Ischemic Attack (TIA)	Brief episode of reduced blood supply to the brain. TIA involving the part of the brain that controls the legs can cause leg paralysis, which is reversible.
Stroke	Permanent blood loss in the brain leading to cell death can lead to stroke and bilateral leg paralysis.
Vitamin B-12 Deficiency	Can cause subacute combined degeneration (SCD) It leads to painless leg paralysis and impaired position/vibration sense.
Multiple Sclerosis	Can present with intermittent painless bilateral leg paralysis, most commonly in middle-aged females.
Tabes Dorsalis	Caused by tertiary syphilis. Progressive sensory ataxia, which can lead to poor coordination.

Since there is no specific diagnostic test, atypical presentations require a high degree of suspicion. Our patient presented with a four-day history of bilateral lower limb weakness with painful swelling and areflexia, which progressed to dysphagia and urinary retention. No preceding infection or trigger made it somewhat atypical, although the absence of antecedent infection does not rule out GBS. Electrolyte derangements and coexisting vitamin deficiencies further masked and delayed the diagnostic process. Avoiding premature closure at the juncture of a vitamin deficiency diagnosis was a key step in the diagnostic reasoning continuum for further investigation into alternative explanations of our patient's acute painful sensory-motor neuropathy. His vitamin deficiencies were not significant enough to stop the diagnostic workup early, prompting the medical team to continue looking for an alternative explanation for his symptoms of pain, swelling, weakness, and incontinence.

GBS should be suspected in any patient presenting with acute and progressive bilateral weakness of limbs accompanied by sensory symptoms, hyporeflexia, and dysautonomia. About 20% of the patients develop respiratory insufficiency, which can be life-threatening, as such continuous cardiopulmonary monitoring is vital in patients with GBS. Erasmus GBS Respiratory Insufficiency Score (EGRIS) is a prognostic tool to assess the requirement for mechanical ventilation after one week of the initial assessment. Atypical presentations include asymmetric involvement or severe and diffuse pain before the onset of weakness. Certain features rule against GBS, such as asymmetric symptoms progressing beyond four weeks and persistent bladder/bowel involvement. According to the National Institute of Neurological Disorders and Stroke (NINDS) criteria, progressive bilateral limb weakness and hyporeflexia are essential to establish the diagnosis. The diagnosis can be supported by CSF examination and electrodiagnostic studies showing albumin-cytological dissociation and polyradiculoneuropathy, respectively. Electrodiagnostic studies are also helpful in differentiating between the variants of GBS [3]. GBS variants lie on a spectrum that often overlaps, depending on the type of nerve fiber involved and the mechanism of injury (demyelination or axonal). Miller-Fischer syndrome (MFS) manifests as ataxia, ophthalmoplegia, and areflexia. Bickerstaff's brainstem encephalitis is a subtype of MFS, presenting with loss of consciousness, paradoxical hyperreflexia, ataxia, and ophthalmoplegia. The pharyngeal-cervical-brachial motor variant presents with pharyngeal, cervical, and brachial muscle group weakness (mimicking botulism). Other variants include pure sensory ataxia, pan-dysautonomia, weakness without sensory signs (pure motor variant), weakness of legs alone (para paretic variant), both sensory and motor symptoms (acute inflammatory demyelinating polyneuropathy (AIDP), acute motor axonal neuropathy (AMAN), and acute motor-sensory axonal neuropathy (AMSAN) [3,8]. AMSAN is thought to be the most severe variant [8,9]. In our patient's case, EMG/NCS confirmed AMSAN, consistent with GBS.

Treatment of GBS requires a multidisciplinary approach. Severe and evolving weakness, autonomic or swallowing difficulty, or an EGRIS score of > 4 warrants an ICU admission. Immunomodulatory therapy should be started when the patient cannot walk independently for more than 10 m or if the symptoms are severe and progressive [3,10]. IVIG works by preventing the activation of macrophages, complement system, and binding of autoantibodies to neuronal targets. The standard dose is 0.4 grams per kilogram of body weight per day for five consecutive days. It is contraindicated in patients with a history of IVIG allergy or immunoglobulin A (IgA) deficiency. Plasma exchange (PE) can also be offered, which removes autoantibodies, cytokines, and other inflammatory molecules, replacing them with albumin or fresh frozen plasma. It is administered over seven to 14 days at a dose of 40-50 ml plasma per kilogram of body weight per session for five sessions. IVIG is preferred over PE due to the ease of availability, cost-effectiveness, and duration of hospital stay [8,11]. Corticosteroids do not have any role in treating GBS [3-7,9,12]. Eculizumab is an emerging therapy with considerable potency, but currently under investigation with only off-label use in GBS. It acts by binding directly to the complement protein C5 thus inhibiting the formation of the membrane attack complex, which causes axonal degeneration [2,11,13].

The frequency and intensity of pain in GBS are highly variable, with most patients complaining during the acute phase of the disease, and this was notable in our patient [1,14]. It may be the initial presenting feature in most patients [15]. Pain in GBS is hypothesized to be multifactorial: (1) large myelinated sensory nerve fiber inflammation (causing hyperesthesia and muscle pain), (2) degeneration of small nerve fibers in the intraepidermal space, (3) nociceptive nerve pain due to radicular nerve root involvement, and (4) different immune antibodies targeting different tissue components causing inflammation and pain [1]. As the pain in GBS may continue up to even a year after diagnosis, management with a multimodal approach merits consideration in drafting a comprehensive treatment plan for these patients [15-17]. Paraesthesia can be treated by pharmacological therapies, such as non-steroidal anti-inflammatory drugs (NSAIDs) and opioids, along with physical intervention such as massage [14]. Our patient had significant hyperalgesia even after acute management with IVIG, causing rehospitalization. Pain in our patient was managed with acetaminophen, gabapentin, carbamazepine, nortriptyline, as well as oxycodone.

Recovery begins in about four weeks for most GBS patients, which may or may not be complete and is often slowed by complications such as nosocomial infections, pressure ulcers, and deep vein thrombosis (DVT). Comprehensive evaluation and monitoring are required for GBS-variant-specific complications, such as dysphagia, urinary retention, corneal ulcerations, etc., as approximately one-third of adult patients will develop permanent residual disability. Post-GBS syndrome refers to the manifestations related to any residual neuropathy after the acute episode of GBS. This manifests as ongoing motor and sensory dysfunction, causing physical debility, chronic pain, contractures, incontinence, etc. [7,8]. Modified Erasmus GBS Outcome Score (mEGOS) can be utilized at admission to determine the patient's probability of regaining ambulatory function [3]. Thus, GBS management warrants a holistic rehabilitation program, as associated complications can pose long-term physical and psychological effects (depression and anxiety) [3].

## Conclusions

Guillain-Barré syndrome is a neurological disorder affecting the peripheral nervous system and presents most often with symmetric weakness, which may be associated with pain or autonomic symptoms. The variability in presenting symptoms and initial laboratory derangements can make diagnosing it quite challenging and if left untreated, it can be fatal. A high level of suspicion is crucial to timely diagnose GBS in cases of acute flaccid paralysis, as early recognition and treatment can hasten recovery. In addition, a comprehensive rehabilitation plan allows for better recovery.
